# Financial Performance of Public Hospitals: A Cross-Sectional Study among Polish Providers

**DOI:** 10.3390/ijerph17072188

**Published:** 2020-03-25

**Authors:** Katarzyna Dubas-Jakóbczyk, Ewa Kocot, Anna Kozieł

**Affiliations:** 1 Health Economic and Social Security Department, Institute of Public Health, Faculty of Health Sciences, Jagiellonian University Medical College, 31-008 Krakow, Poland; ewa.kocot@uj.edu.pl; 2 Senior Health Specialist, Health, Nutrition & Population, World Bank, The World Bank Office in Poland, 00-113 Warsaw, Poland; akoziel@worldbank.org

**Keywords:** public hospitals, financial performance, liabilities, financial deficit

## Abstract

There is growing evidence of a positive association between health care providers’ financial standing and the quality of care. In Poland, the instable financial situation and growing debt of public hospitals has been a source of concern for more than two decades now. The objectives of this paper were to compare the financial performance of public hospitals in Poland, depending on the ownership and organizational form; and analyze whether there is an association between financial performance and the chosen variables. We conducted a cross sectional study covering the whole population of public hospitals operating in 2018. The total number of included units was 805. The hospitals’ financial outcomes were measured by several variables; Spearman’s rank correlation was calculated, and a multivariable logistic regression model was performed. In 2018, the majority of public hospitals in Poland (52%) generated a gross loss, while 40% hospitals had overdue liabilities. There were statistically significant differences between hospital groups, with university hospitals and those owned by counties (local hospitals) being in the most disadvantageous situation. Additionally, corporatized public hospitals performed worse than those functioning in the classic legal form of independent health care units. Urgent actions are needed to measure and monitor the potential impact of financial performance on the quality of care.

## 1. Introduction

There is growing evidence on the positive association between the health care providers’ financial standing and the quality of care [1,2,3,4,5,6]. In general, providers that generate profit might have better capacity to finance investment and pay higher wages and/or attract more skilled staff, contributing to quality of care improvement [5]. Financially stable hospitals are better able to maintain reliable systems and provide resources for quality improvement [1]. Therefore, monitoring financial performance of heath care units is important in the context of improving efficiency and securing the organizational sustainability of care provision [7,8].

In Europe, hospitals constitute the cornerstone of health care provision and are financed mainly from public sources [9]. In 2017, services provided by hospitals consumed more than 35% of total current health expenditures in 24 European countries (out of the 33 for which data are available); the share of public financing in total hospital expenditures was more than 80% in 26 countries and publicly owned hospital beds constituted more than 60% of all hospital beds in 22 countries (out of the 31 for which data are available) [10]. Within the last three decades, implementation of diverse hospital care cost-containment mechanisms has been a common trend of European health systems [9,11].

In Poland, as in many other Central and Eastern Europe countries, the hospital sector is characterized by historically oversized infrastructure, the prevalence of publicly owned hospitals/beds, and a highly fragmented hospital ownership structure [12]. In 2017, there were 6.6 hospital beds per 1000 people in Poland, in comparison to the EU average of 5.0 (in case of curative care beds the ratios were 4.9 vs. 3.7) [10]. In 2018, publicly owned hospital beds constituted 87% of the total number of hospital beds in Poland [13]. Private hospitals, although numerous, are usually small, often single-specialty units. The ownership structure of public hospitals is fragmented, divided between the three levels of local government (municipalities, counties, voivodships), ministries, and medical universities [14]. Regardless of the ownership structure, the vast majority of services provided by hospitals are financed from public sources (95% in 2016–2017) [15]. The main payer is the National Health Fund, whose main revenue sources are premiums from the public health insurance system. The payer contracts medical services based on a tender or (rarely) negotiations formula and is obliged to guarantee equal principles for all types of providers [16]. The hospital owners often provide financial support for investments project in their hospitals [14], yet can be characterized by different capacities in this area [17].

The instable financial situation and growing debt of public hospitals in Poland has been a source of concern for more than two decades now [18,19,20,21,22]. Numerous hospitals face challenges in setting their liabilities while their owners (local governments, medical universities, and ministers) are forced to secure additional sources of financing. The Ministry of Health systematically gathers and publishes aggregated data on the total value of hospitals liabilities, including overdue ones (arrears) [23]. The main share of hospital arrears goes to drugs, materials, and energy suppliers (70% of total overdue liabilities in 2018) [23]. Running arrears usually do not impact service delivery—vendors usually agree to renegotiate the payment schedule/interest rate. The dominant legal form of public hospitals (SPZOZ) has no bankruptcy capacity, which may contribute to more flexible vendor–buyer relations as well as less strict financial accountability policies [20]. However, there have been isolated cases when the bailiff has taken over the indebted hospital account [24]. Additionally, the persistence of arrears generates additional costs—penalty interests for not settling liabilities on time. Analysis on a group of 52 hospitals owned by local government units showed that in a two year period, 2015–2016, the aggregated value of such penalties was more than 39 million PLN, approx. 5% of the total arrears value [22]. Additionally, the debt restructuration processes carried out by hospitals might lead to increasing the share of long-term liabilities, e.g., by taking loans and opening lines of credit in banks or other financial intermediaries in order to settle arrears (the debt roll-over process). The fragmented data for a group of 52 hospitals owned by local governments show that between 2015 and 2016, approx. 13% of the total value of loans was devoted to settling previously generated liabilities. The loan security usually consisted of the revenues from the contract with the public payer and/or securities provided by the local governments [22]. As a consequence, the persistence of the arrears generates a significant financial burden for the hospital owners, increasing their risk of insolvency. Within the last two decades the central government has made several direct attempts to deal with the hospital debts problem. They have provided different requirements and solutions for indebted hospitals: bail-out programs including restructuration elements (2005) [25] or corporatization with debt-relief plans (2009 and 2011) [26,27]. Additionally, also implemented in 2017, the hospital network reform, although not directly targeted at the hospital’s debt problem, included elements aimed at improving the relationship between the payer and hospital care providers while ensuring continuity and stability of financing for the latter [28].

Several recently published studies have been focused on financial performance of hospitals in Poland [29,30,31]. Yet these covered a limited number of hospitals, based on their specialty and/or geographical location. For example, a study by Siedlecki et al. (2016) covered 201 hospitals and included data from 2012. The authors compared financial indicators between urban and rural hospitals, concluding that the latter are characterized by better financial condition [29]. Krzeczewski et al. (2019) conducted a longitudinal study (2007–2016) on a group of 118 hospitals, comparing financial performance of units from big vs. smaller cities. The results indicated that hospitals located in cities with a population above 100,000 people perform better financially than those from smaller cities [31]. The author of this paper published a study on financial conditions of university hospitals in Poland [30]. The study covered 21 out of 41 university hospitals functioning in 2014. The results indicated the general difficult financial situation of the analyzed units, yet with simultaneous huge disparities between individual providers [30]. Additionally, the available reports developed by audit institutions present individual hospitals case studies and/or include a small sample of hospitals [20,22,32,33]. For example, a longitudinal (2005–2014) analysis conducted by the Supreme Audit Office (2016) covered eight public hospitals [20], which received mentioned in the previous paragraph government financial support aimed at managing the hospitals debt problem [25]. The results showed, that only in three out of eight audited hospitals, did the support received lead to lasting improvement of the financial situation, including the complete reduction of overdue liabilities [20].

The general objective of the current analyses was to provide a comprehensive overview of the financial standing of all public hospitals in Poland in 2018. The specific objectives were (1) to compare the financial performance of public hospitals depending on the ownership status and the type of the organizational form; (2) to analyze whether there is an association between the public hospitals’ financial standing and the chosen variables, e.g., total assets, revenues, and costs. 

## 2. Materials and Methods

### 2.1. Study Design and Participants

We conducted a cross sectional study covering the whole population of public hospitals operating in 2018 in Poland. These hospitals can take three legal/organizational forms: (1) the basic legal form of independent health care units (samodzielny publiczny zakład opieki zdrowotnej—SPZOZ), (2) research institutes, and (3) corporatized public hospitals (Table 1). SPZOZs constitute the vast majority (both in terms of number of units and their share in the total number of beds). Both SPZOZs and research institutes do not have bankruptcy capacity and the final responsibility for their financial deficits falls on the owners. The type of owner roughly corresponds with the scope of services provided: counties usually own general local hospitals, voivodeships own regional, multidisciplinary hospitals while medical universities run highly specialized clinics [14]. Hospitals owned by ministries constitute a diverse group, including, inter alia: single specialty clinics, military hospitals, and highly specialized research institutes.

The total number of public hospitals for which data were available in 2018 was 805 (95% of all public hospitals). This number included 659 SPZOZs, 17 research institutes, and 129 corporatized public hospitals. The information on the hospital owner was available only for the first two categories, thus, while comparing hospitals per ownership and organizational form, a total number of 676 and 805 units were included, respectively. 

### 2.2. Data Sources and Variables

The source of data was the statistics form ‘MZ-03,’ submitted by all public health care providers to the Center for Information Systems in Health Care (an analytical institution supervised by the Ministry of Health). The form covers a range of financial data retrieved mainly from the balance sheet and the profit and loss statement [34]. The form submission is obligatory for all public health care providers in Poland (the response rate each year is above 95%).

For the providers financial condition assessment two categorical and three continuous variables were used. The categorical variables were:

- generation/existence of gross profit (profit before tax); yes—there is gross profit, no—there is no gross profit, the hospital generated gross loss
- generation/existence of arrears (overdue liabilities); yes—there are overdue liabilities, no—there are no overdue liabilities

In terms of continuous variables, the following outcome indicators (measured in percentages) were used:

- gross profit margin (profit or loss before tax per total revenues);
- debt ratio (total liabilities per total assets);
- the share of arrears (overdue liabilities) in total liabilities.

The choice of outcome indicators was based on (1) the data availability, (2) their broad applicability in financial analyses [7,35,36], and (3) their particular importance for the Polish health sector (indicators involving arrears value).

### 2.3. Statistical Analyses

Continuous variables were expressed as mean (standard deviation, SD) or as median (interquartile range, q1–q3), as appropriate. The Shapiro–Wilk test was used to assess conformity with a normal distribution. The Kruskal–Wallis test was used to compare continuous variables between the ownership and organizational form groups. Categorical variables were described by percentages and compared using the χ2 test. 

Spearman’s rank correlation was calculated to assess the association between the chosen financial variables. A univariate and multivariable logistic regression model was performed in order to identify the significant predictors of gross profit generation and arrears existence (as dichotomous variables). Odds ratios (ORs) with their corresponding 95% confidence intervals (CI) were computed and reported. Diagnostic accuracy was assessed using receiver operating characteristics (ROC) curves, and areas under the ROC curve (AUC) with 95% CIs. Statistical analyses were performed using SPSS 23.0 (SPSS Inc., Chicago, IL, USA). *p*-values < 0.05 were accepted as statistically significant. 

## 3. Results

### 3.1. Participants’ Descriptive Statistics

There are huge disparities between the analyzed units in terms the values of the main financial variables. Table 2 presents median values of the total assets, revenues, costs, and liabilities as well as the gross profit/loss and arrears per analyzed hospitals groups. Tables S1 and S2 in Supplementary online files present complete descriptive statistics. In 2018, among all 805 public hospitals, the median values (q1–q3) for the total assets, revenues, costs, and liabilities were as follows: 27.9 (9.8–68.3) million PLN; 35.8 (12.8–81.1) million PLN; 36.5 (12.9–84.5) million PLN; 6.4 (1.4–20.9) million PLN. The median value of the gross loss was −0.6 (−2.8–0.3) million PLN, while in terms of arrears, the median was above zero only in the case or research institutes (Table 2). If applying the value of total assets and/or revenues as a proxy measure of the hospital size, the data confirm that county hospitals are usually small (local) hospitals; those owned by voivodeship are of medium size; while university clinics include the biggest units. Hospitals owned by ministers constitute the most differential, in terms of the unit size, group (Table 2, Tables S1 and S2 in Supplementary Files). 

### 3.2. Financial Performance per Hospital Owners

**Figure 1:**
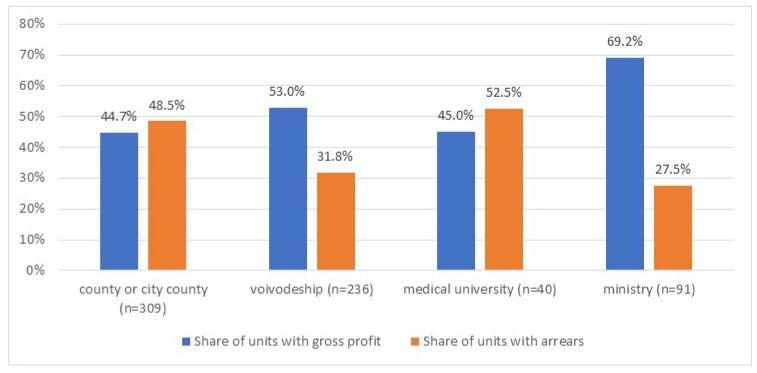
Share of hospitals with gross profit and arrears (%) per ownership groups in 2018. (*p*-values from *χ*^2^ test; *p*-value < 0.001 for both categorical variables.)

In 2018, out of 676 public hospitals (for which information on the owner was available), only 344 (50.9%) providers generated a gross profit, while 272 (40.1%) hospitals had arrears. The share of units with a gross profit ranged from 44.7% among county hospitals to 69.2% for those owned by ministries (*p* < 0.001) (Figure 1). In the case of two owners (counties and medical universities) the majority of units generated a gross loss. The biggest share of units with arrears was in the group of providers owned by medical universities: 52.5%, while the lowest was among ministerial units: 27.5% (*p* < 0.001). 

In 2018, there were statistically significant differences in the values of the all three financial performance indicators between the unit ownership groups (Table 3). In the case of the gross profit margin ratio, the median value was the highest in providers owned by ministries 1.0% (−1.3%–3.6%) and the lowest in county providers −1.2% (−5.6%–1.2%). The debt ratio was the highest in university hospitals—median value of 30.6% (19.1%–51.5%). Additionally, the median value of the share of arrears in total liabilities, although it equals 0.0% for all ownership groups, has the broadest interquartile (q1–q3) range in the case of university hospitals. In general, university hospitals and those owned by counties have the most disadvantageous values of the three outcome indicators (Table 3).

Post hoc pairs comparison indicated statistically significant differences inter alia between (1) ministerial hospitals and those owned by counties (*p* < 0.001) and voivodeships (*p* < 0.001) in terms of the gross profit margin median value; (2) voivodeship hospitals and those owned by counties (*p* > 0.001); medical universities (*p* < 0.05) and ministries (*p* < 0.05) in terms of the debt ratio; (3) county hospitals and those owned by voivodeships (*p* < 0.05) and medical universities (*p* < 0.05) in terms of the share of arrears in total liabilities (Table 3).

### 3.3. Financial Performance per Hospital Organizational Form

**Figure 2:**
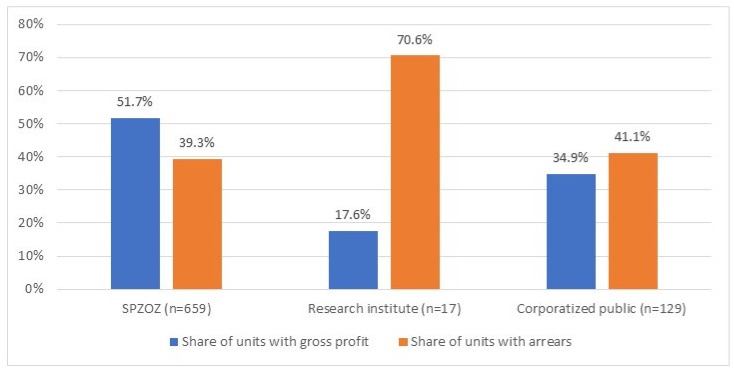
Share of hospitals with gross profit and arrears (%) per organizational form, in 2018. (*p*-values from *χ*^2^ test; *p*-value < 0.001 for both categorical variables.)

In 2018, out of 805 public hospitals only 389 (48.3%) providers generated a gross profit, while 324 (40.2%) hospitals had arrears. The share of units with a gross profit ranged from 17.6% among research institutes to 51.7% for SPZOZs (*p* < 0.001) (Figure 2). In the case of two organizational forms (research institutes and corporatized public hospitals) the majority of units generated a gross loss. The share of units with arrears ranged from 39.3% among SPZOZs to 70.6% among research institutes (*p* < 0.001). 

In 2018, there were statistically significant differences in the values of the all three outcome measures between the units with different organizational form (Table 4). SPZOZs have the most advantageous values of all three indicators: the highest median value of the gross profit margin: 0.0% (−4.0%–1.4%) and the lowest median value of the debt ratio: 19.6% (10.6%–39.8%), as well as the lowest interquartile range (q1–q3) of the share of arrears in total liabilities: 0.0% (0.0%–5.6%) (*p* < 0.001). Research institutes had the higher share of arrears in total liabilities among the three provider groups: 6.5% (0.0%–20.4%) (*p* < 0.001). Post hoc pairs comparison indicated statistically significant differences between SPZOZs and corporatized public hospitals in terms of both the gross profit margin (*p* < 0.001) and debt ratio median values (*p* < 0.05) (Table 4). Additionally, research institutes had a higher median value of the share of arrears in total liabilities than hospitals functioning in both remaining organizational forms (*p* < 0.05) (Table 4). 

### 3.4. Factors Associated with Hospitals’ Financial Performance

As expected, there is strong positive correlation between a hospital’s total assets and the value of both total costs and revenues (r = 0.92, *p* < 0.001 for both variables) (Table S3 in Supplementary Material). All three variables which can be used as proxy indicators of the hospital’s size (total assets, revenues, and costs) are positively correlated with the value of arrears and the debt ratio, and negatively correlated with the gross profit margin. 

Results of the logistic regression model confirm that both the ownership status and the organizational form, as well as the hospital size (measured by the value of total assets/revenues/costs) can be statistically significant predictors of the hospital’s financial standing measured by the generation of gross profit and existence of arrears. Supplementary files present results of univariate (Tables S4 and S5) and multivariable logistic regression models (Tables S6–S9). In terms of the latter, when including the ownership status variable, the increase in the value of total revenues by 10 million PLN, decreases the chances of gross profit generation by 7% (OR = 0.93, 95% CI 0.90–0.95, *p* < 0.001) and increases the chances of arrears by 8% (OR = 1.08, 95% CI 1.05–1.10, *p* < 0.001) (Table 5). When including the organizational form variable, an increase in the value of total revenues by 10 million PLN decreases the chances of gross profit generation by 6% (OR = 0.94, 95% CI 0.92–0.96, *p* < 0.001) and also increases the chances of arrears existence by 6% (OR = 1.06, 95% CI 1.04–1.08, *p* < 0.001) (Table 6). Corporatized public hospitals are 54% less likely to generate gross profit than SPZOZs (OR = 0.46, 95% CI 0.31–0.68, *p* < 0.001). Similar associations can be observed in multivariable logistic regression models, when including total assets or cost variables (Tables S6–S9 in Supplementary Material). 

## 4. Discussion

In 2018, out of 805 public hospitals in Poland, the majority of units (59.8%) generated a gross loss, while 40.2% of hospitals had arrears (overdue liabilities). There were statistically significant differences in the hospitals’ financial performance depending on the type of owner (Figure 1, Table 3) and the legal form (Figure 2, Table 4). Local, county hospitals and university clinics are characterized by more disadvantageous values of the financial situation indicators than regional hospitals and those owned by ministries (Table 3). Additionally, hospitals functioning in the prevailing, classic legal form of independent health care units (SPZOZ) perform better financially than corporatized public hospitals and research institutes (Figure 2, Table 4). 

Numerous previous studies, conducted in different countries, indicated that the type of ownership might be an important factor determining hospital financial performance [8,37,38]. Our study focused solely on public hospitals, thus, the scope of owners was limited. Still, statistically significant differences in the hospital financial performance were identified. Currently in Poland, local (county and/or city-county) hospitals are facing major challenges related to both the level of financing as well as doctor deficits [39,40]. In 2018, the majority of county hospitals generated a gross loss (55.3%) while almost half of units had overdue liabilities (48.5%). Between 2018 and 2019, the National Association of County Hospitals (including more than 130 county hospitals) issued several official petitions to the central policy-makers, asking for increased financing and emphasizing their risk of insolvency, mainly due to rising staff costs and limited financing under the new 2017 network regulations [41]. In general, the problem of small county hospitals functioning and their role in the national health system has been a central issue of policy-makers’ debates for many years now. There have been proposals of their acquisition by regional administrations, liquidation, and/or transformation into local long-term care centers [21]. This type of solution was introduced, inter alia, in Romania, Hungary, and Croatia [12]. 

University hospitals constitute a unique group of in-patient services providers. They provide highly specialized care for most complicated, severe cases, and also conduct teaching and research activities. Challenges related to developing adequate payment mechanisms for teaching hospitals have been addressed by researchers from different countries [42,43,44]. In Poland, the majority of university hospitals operating in 2018 generated a gross loss and had overdue liabilities (55% and 53% of units, respectively). A mixture of factors, including lack of dedicated regulations taking into account university hospitals’ special character (high cost of services, the burden of educational activities), weak governance, and a unique legal environment which hampers flexible management approaches, might contribute to the fact that many university hospitals are among the highly indebted ones [30,45]. 

Our results showed statistically significant differences in financial performance outcomes between hospitals functioning in different organizational/legal forms (Figure 2, Table 4). For example, corporatized hospitals had a more disadvantageous median value of gross profit/loss margin and a higher debt ratio than units functioning in the classical legal form of independent health care units (−2.1% (−9.1%–0.9%) vs. 0.0% (−4.0%–1.4%); *p* < 0.001 and 28.0% (13.5%–48.3%) vs. 19.6% (10.6%–39.8%); *p* < 0.05, respectively) (Table 4). However, in both hospital groups, there was high diversity of the individual units’ financial performance. Corporatization of public hospitals was pursued in many countries as a means to improve hospital management, i.e., by increasing autonomy [9,46]. In Poland, this process was based on the individual, bottom-up initiatives of the hospital owners (mainly local governments) and supported by two central government programs conducted between 2009 and 2013 [26,27]. In general, under both programs, owners that decided to transform their hospitals into commercial code companies might receive additional financial support from the state to cover existing hospital liabilities (a corporatized unit started operation debt free). Our results are in line with some of the previously published analyses [32] and suggest that corporatization of public hospitals have not improved their financial standing. 

Finally, regarding factors that might contribute to a hospital’s difficult financial situation, our results showed a positive correlation between the hospital’s size (measured, i.a., by total assets value) and the debt ratio and negative correlation with the gross profit margin. This contradicts the existence of economies of scale, yet, can be explained by the overall characteristics of the Polish hospital sector. University clinics and research institutes constitute the group of the biggest hospitals in Poland. The majority are highly specialized, multidisciplinary centers with several hundred beds. The issue of the difficult financial situation of this type of hospitals has been emphasized, i.a., in the analyses and case-studies conducted by the Supreme Audit Office [33,45,47,48]. The auditors indicated that these hospitals face numerous challenges related to ineffective management and inadequate revenue sources [47,48].

Hospitals throughout the world operate in heavily regulated environments [49,50]. There is an abundance of external factors that influence different aspects of hospital functioning, including the ability to generate revenues and contain costs. Therefore, while assessing hospitals’ financial performance, the national health policy context must be carefully analyzed. In Poland, numerous previous analyses indicated that there is no single, leading cause of public hospitals’ poor financial standing and debt problem [18,19,20,21,22,39]. Instead there is a mix of both macro- and micro-level factors that contribute to undermining public hospitals’ financial standing. At the system level, these include weak stewardship [18,19]; oversized infrastructure [19]; inadequate tariff valuation [19,21]; central regulations on medical workers’ salaries [20,39]; underfunding and/or lack of health needs analysis and matching funding; and inadequate financial mechanisms [18,19]. At the level of particular hospitals, additional micro-level features might be leading factors, including poor management (i.a., regarding cost containment procedures, especially in relation to staff costs) [20]; weak owner control [19]; the general state of the hospital’s infrastructure (the need for investment expenditures) [19]. The importance of individual micro-factors is highlighted by a huge diversity in hospitals’ financial standings. Entities with a gross loss and a high debt level as well as profitable ones with proper liquidity coexist [20,30]. 

The problem of public hospitals running overdue liabilities exists in many Central and Eastern European countries (e.g., Romania, Hungary, Croatia, and Bulgaria) [12]. However, there seems to be a gap in literature concerning financial conditions of hospital providers in Europe. The vast majority of the relevant studies identified by the authors of this paper, are focused on the United States (US) market [1,2,3,4,5,6,7,35,37,38]. To the authors knowledge, this is the first study comprehensively assessing public hospitals’ financial standing in Poland based on national level data. The whole population of public hospitals was covered, including data usually excluded from publicly available statistical bulletins [15,51] on ministerial hospitals, research institutes, and corporatized public hospitals. The study provides an important insight for national policy-makers. There are, however, important limitations to be noted. Our data source (form MZ-03) provides only limited financial data [34], thus, we could not include more detailed characteristics of hospitals (e.g., some basic input data: number of beds/wards, number of staff or type of specialty, reference level). As a consequence, we could not include in our analyses other important variables that could potentially influence hospitals’ financial performance. Additionally, no data on hospital outputs were available. In general, in Poland the availability of data on health system providers functioning constitutes a huge challenge [52]. In the case of the hospital sector, there is no comprehensive data warehouse allowing for an individual unit comparison [52]. As a consequence, we could not include more precise hospital characteristics in our analyses, or assess the relationship between hospitals’ financial standing and any form of outputs (e.g., number of discharges, beds-days, etc.). Another important limitation is related to the choice of the outcome indicators. The concept of ‘financial performance/condition’ is complex and multidimensional [1,35]. Therefore, the choice of the indicators may impact the results. Recent evidence from the US suggests that composite financial performance scores (combining multiple measures) might be more appropriate for hospitals than single indicators [1]. Due to the data availability, our study covered a limited number of five (partially interrelated) financial performance indicators.

Being aware of the above-mentioned limitations, we believe that this study provides important implications for both researchers and policy-makers. In the case of the former, there is a need to plan and conduct studies aimed at (1) developing a comprehensive framework/composite scores for hospitals financial performance assessment; and (2) assessing the potential impact of financial performance of public hospitals in Poland on the quality of care provided. The available evidence suggest that hospitals’ strong financial performance is associated with an improved quality of care [1,3,4,5,6]. Thus, taking into account the results of our study, conducting studies aimed at measuring the relationship between hospitals’ financial standing and the quality of care provided is extremely important in Poland. In the case of implications for national policy-makers, the most important ones relate to (1) developing regulations aimed at providing access to data allowing for above-mentioned research; and (2) close monitoring of the fiscal context of the persistence of public hospitals’ arrears [53,54]. In case of the latter, the impact of hospitals’ debts on the overall public deficit in Poland should be analyzed.

## 5. Conclusions

In 2018, the majority of public hospitals in Poland (52%) generated a gross loss, while 40% of hospitals had overdue liabilities. There were statistically significant differences between hospital groups, with university hospitals and those owned by counties (local hospitals) being in the most disadvantageous financial situation (Table 3). Additionally, corporatized public hospitals performed worse than those functioning in the classic legal form of independent health care units (Table 4). There is an urgent need to measure and monitor the potential impact of the financial performance of public hospitals in Poland on the quality of care provided.

## Figures and Tables

**Table 1:** Legal/organizational forms of public hospitals in Poland.

Legal Form	Legal Consequences of Financial Deficit	Owners	Share of Units in Total Number of Public Hospitals Beds in 2018^c^
Independent health care units (samodzielny publiczny zakład opieki zdrowotnej—SPZOZ)	• Do not have bankruptcy capacity; • If the hospital cannot cover the financial loss^a^ the owner has to cover it or make a decision about hospital liquidation^b^	• Local governments (counties/cities; voivodships) • Medical universities • Ministries	80.2% (per owner: 29.9% counties/cities; 34.5% voivodeships; 11.5% medical universities; 4.3% ministries)
Research institutes	• Do not have bankruptcy capacity; • In case of liquidation the liabilities are taken over by the state	• Ministry of Health (supervisory body)	3.5%
Corporatized public hospitals (commercial companies with the majority of/all shares belonging to public body)	• Subject to regulations applicable to commercial code companies (incl. bankruptcy capacity)	• Local governments (counties/cities; voivodships)	16.3%

^a^ the regulations related to the situation wherein the hospital generates a financial loss (calculated as net loss + depreciation costs) were changed over time (until 2015, the owner could also make the decision to corporatize the indebted hospital). ^b^ the decision to liquidate is not straightforward, especially in the case of local governments as they are still obliged to guarantee the continuity of service provision for the local community. ^c^ publicly owned hospital beds constituted 87% of the total number of hospital beds.

**Table 2:** Median values of basic financial variables per hospital groups (million PLN, 2018).

Hospitals Classification:		*n*	Median (q1–q3), Million PLN					
			Total Assets	Total Revenues	Total Costs	Gross Profit/Loss	Total Liabilities	Arrears
**Owner**	County or city County	309	24.7 (7.7–49.0)	36.5 (11.9–62.7)	37.4 (11.9–63.8)	−0.2 (−2.4–0.2)	8.0 (1.4–17.9)	0.0 (0.0–1.5)
	Voivodeship	236	34.0 (10.8–82.4)	36.8 (12.3–121.2)	36.2 (12.2–121.4)	0.4 (−2.6–0.3)	5.6 (1.2–26.3)	0.0 (0.0–0.06)
	Medical university	40	138.2 (81.6–211.3)	224.4 (115.1–353.8)	231.6 (117.5–358.7)	−0.5 (−6.9–0.7)	42.6 (16.4–85.6)	0.0 (0.0–11.7)
	Ministry	91	22.2 (6.1–80.4)	19.3 (6.6–60.8)	18.7 (6.3–65.5)	0.9 (−0.6–0.4)	2.3 (0.5–12.2)	0.0 (0.0–0.0)
**ALL owners**		**676**	**28.4 (9.4–70.2)**	**36.3 (12.1–87.3)**	**36.6 (12.2–89.8)**	**1.4 (−2.6–0.3)**	**6.7 (1.3–22.6)**	**0.0 (0.0–1.0)**
**Legal form**	SPZOZ	659	27.9 (9.2–68.3)	35.9 (11.5–85.6)	35.9 (11.5–86.8)	0.0 (−2.4–0.3)	6.4 (1.2–21.3)	0.0 (0.0–0.9)
	Research institute	17	141.9 (34.2–282.8)	133.9 (35.0–281.2)	137.8 (35.5–284.8)	−3.1 (−7.3–(−0.7))	40.7 (6.5–92.4)	4.3 (0.0–11.6)
	Corporatized public	129	26.0 (10.7–50.2)	33.9 (20.1–60.3)	35.0 (21.3–61.6)	−0.7 (−3.7–0.2)	5.9 (2.9–14.7)	0.0 (0.0–0.9)
**ALL legal forms**		**805**	**27.9 (9.8–68.2)**	**35.8 (12.8–81.1)**	**36.5 (12.9–84.5)**	**−0.1 (−2.8–0.3)**	**6.4 (1.4–20.9)**	**0.0 (0.0–1.0)**

**Table 3:** Financial indicators per ownership group, in 2018.

Variable (Percentage, Median, q1-q3)/Owner	‘A’ County or City County	‘B’ Voivodeship	‘C’ Medical University	‘D’ Ministry	H-Value	*p*-Value
*n*	309	236	40	91		
Gross profit margin	−1.2 (−5.6–1.2)	0.0 (−4.1–0.9)	−0.7 (−2.8–0.4)	1.0 (−1.3–3.6)	23.3	<0.001
Debt ratio	24.5 (14.2–51.2)	17.9 (9.9–33.7)	30.6 (19.1–51.5)	9.8 (5.1–19.5)	60.9	<0.001
Share of arrears in total liabilities	0.0 (0.0–10.4)	0.0 (0.0–2.3)	0.0 (0.0–13.6)	0.0 (0.0–0.1)	22.3	<0.001

Median (q1–q3); H-value of the Kruskal–Wallis test statistic; *p*-values from the Kruskal–Wallis test; statistically significant post hoc pairs comparisons (* *p* < 0.05; ** *p* < 0.001): gross profit margin (A-D **, B-D **); debt ratio (A-B **, A-D **, B-C *, B-D *, C-D **); share of arrears in total liabilities (A-B *, A-D *, C-D *).

**Table 4:** Financial indicators per organizational form comparison in 2018.

Variable (Percentage, Median, q1-q3)/Organizational Form	‘A’ SPZOZ	‘B’ Research Institute	‘C’ Corporatized Public	H-Value	*p*-Value
*n*	659	17	129		
Gross profit margin	0.0 (−4.0–1.4)	−2.5 (−7.9– (−0.4))	−2.1 (−9.1–0.9)	17.2	<0.001
Debt ratio	19.6 (10.6–39.8)	26.1 (15.1–73.5)	28.0 (13.5–48.3)	9.3	0.01
Share of arrears in total liabilities	0.0 (0.0–5.6)	6.5 (0.0–20.4)	0.0 (0.0–7.8)	9.2	0.01

Median (q1–q3); H-value of the Kruskal–Wallis test statistic; *p*-values from Kruskal–Wallis test; statistically significant post hoc pairs comparisons (* *p* < 0.05; ** *p* < 0.001): gross profit margin (A-C **); debt ratio (A-C*); share of arrears in total liabilities (A-B *, B-C *).

**Table 5:** Multivariable logistic regression models predicting gross profit and arrears generation (ownership group and revenues as variables).

Variable	Generation/Existence of Gross Profit		Generation/Existence of Arrears	
	Adjusted OR (95% CI)	*p*-Value	Adjusted OR (95% CI)	*p*-Value
**Ownership group:**				
Country or city	1		1	
Voivodeship	1.73 (1.21–2.47)	0.003	0.38 (0.26–0.55)	<0.001
Medical university	4.05 (1.74–9.46)	<0.001	0.30 (0.13–0.70)	0.005
Ministry	3.29 (1.94–5.60)	<0.001	0.34 (0.20–0.58)	<0.001
**Revenues (10 million PLN)**	0.93 (0.90–0.95)	<0.001	1.08 (1.05–1.10)	<0.001
**AUC**	0.70 (0.66–0.74)		0.71 (0.67–0.75)	

OR—odds ratio, AUC—area under the curve.

**Table 6:** Multivariable logistic regression models predicting gross profit and arrears generation (organizational form and revenues as variables).

Variable	Generation/Existence of Gross Profit		Generation/Existence of Arrears	
	Adjusted OR (95% CI)	*p*-value	Adjusted OR (95% CI)	*p*-Value
**Organizational form:**				
SPZOZ	1		1	
Research institute	0.31 (0.08–1.13)	0.077	2.31 (0.75–7.09)	0.144
Corporatized public	0.46 (0.31–0.68)	<0.001	1.17 (0.79–1.73)	0.446
**Revenues (10 million PLN)**	0.94 (0.92–0.96)	<0.001	1.06 (1.04–1.08)	<0.001
**AUC**	0.69 (0.65–0.72)		0.71 (0.67–0.74)	

OR—odds ratio, AUC—area under the curve.

## Supplementary Materials

The following are available online at https://www.mdpi.com/1660-4601/17/7/2188/s1, Table S1: Hospitals’ descriptive statistics per ownership group, Table S2: Hospitals’ descriptive statistics per organizational form, Table S3: Univariate logistic regression models predicting gross profit and arrears generation (including ownership groups), Table S4: Univariate logistic regression models predicting gross profit and arrears generation (including organizational form groups), Table S5: Spearman correlation coefficients (*n* = 805), Table S6: Multivariable logistic regression models predicting gross profit and arrears generation (ownership group and total assets as variables), Table S7: Multivariable logistic regression models predicting gross profit and arrears generation (organizational form and total assets as variables), Table S8: Multivariable logistic regression models predicting gross profit and arrears generation (ownership group and total costs as variables), Table S9: Multivariable logistic regression models predicting gross profit and arrears generation (organizational form and total costs as variables).
