# Supplementary material for: Financial Performance of Public Hospitals: A Cross-Sectional Study among Polish Providers
Source: Int J Environ Res Public Health. 2020 Mar 25;17(7):2188. doi: 10.3390/ijerph17072188 (PMC7177959; doi:10.3390/ijerph17072188)
Supplement: Supplementary file 1 [file ijerph-17-02188-s001.pdf]

Table 1. Hospitals' descriptive statistics per ownership group.

Hospitals classification	n	Total assets	Total revenues	Total costs	Gross profit/loss	Total liabilities	Arrears
			Median (q1-q3) (Million PLN)				
County or city county	309	24.7 (7.7–49.0)	36.5 (11.9–62.7)	37.4 (11.9–63.8)	-0.2 (-2.4–0.2)	8.0 (1.4–17.9)	0.0 (0.0–1.5)
Voivodeship	236	34.0 (10.8–82.4)	36.8 (12.3–121.2)	36.2 (12.2–121.4)	0.4 (-2.6–0.3)	5.6 (1.2–26.3)	0.0 (0.0–0.06)
Medical university	40	138.2 (81.6–211.3)	224.4 (115.1–353.8)	231.6 (117.5–358.7)	-0.5 (-6.9–0.7)	42.6 (16.4–85.6)	0.0 (0.0–11.7)
Ministry	91	22.2 (6.1–80.4)	19.3 (6.6–60.8)	18.7 (6.3–65.5)	0.9 (-0.6–0.4)	2.3 (0.5–12.2)	0.0 (0.0–0.0)
**Total**	**676**	**28.4 (9.4–70.2)**	**36.3 (12.1–87.3)**	**36.6 (12.2–89.8)**	**1.4 (-2.6–0.3)**	**6.7 (1.3–22.6)**	**0.0 (0.0–1.0)**
			Average (min–max) (Million PLN)				
County or city county	309	36.9 (0.2–414.0)	45.9 (0.4–306.7)	47.5 (0.4–356.3)	-1.6 (-49.6–6.2)	14.4 (0.0–422.1)	1.7 (0.0–63.7)
Voivodeship	236	65.4 (0.9–477.4)	75.1 (1.6–480.9)	77.6 (1.5–503.0)	-2.5 (-35.2–16.4)	21.0 (0.1–321.3)	1.9 (0.0–56.7)
Medical university	40	155.5 (7.0–595.5)	248.7 (2.2–835.8)	252.2 (2.2–849.6)	-3.5 (-23.6–21.6)	73.0 (0.3–389.3)	13.3 (0.0–128.9)
Ministry	91	80.0 (0.1–1146.0)	70.1 (0.1–1285.8)	71.0 (0.6–1318.4)	-1.0 (-32.6–6.0)	20.8 (0.0–355.1)	2.3 (0.0–55.6)
**Total**	**676**	**59.6 (0.1–1146.0)**	**71.4 (0.1–1285.8)**	**73.3 (0.4–1318.4)**	**-1.9 (-49.6–21.6)**	**21.0 (0.0–422.1)**	**2.5 (0.0–128.9)**

Table 2. Hospitals' descriptive statistics per organizational form.

Hospitals classification	n	Total assets	Total revenues	Total costs	Gross profit/loss	Total liabilities	Arrears
			Median (q1-q3) (Million PLN)				
SPZOZ	659	27.9 (9.2–68.3)	35.9 (11.5–85.6)	35.9 (11.5–86.8)	0.0 (-2.4–0.3)	6.4 (1.2–21.3)	0.0 (0.0–0.9)
Research institute	17	141.9 (34.2–282.8)	133.9 (35.0–281.2)	137.8 (35.5–284.8)	-3.1 (-7.3– (-0.7))	40.7 (6.5–92.4)	4.3 (0.0–11.6)
Corporatized public	129	26.0 (10.7–50.2)	33.9 (20.1–60.3)	35.0 (21.3–61.6)	-0.7 (-3.7–0.2)	5.9 (2.9–14.7)	0.0 (0.0–0.9)
**Total**	**805**	**27.9 (9.8–68.2)**	**35.8 (12.8–81.1)**	**36.5 (12.9–84.5)**	**-0.1 (-2.8–0.3)**	**6.4 (1.4–20.9)**	**0.0 (0.0–1.0)**
			Average (min - max) (Million PLN)				
SPZOZ	659	55.3 (0.1–595.5)	67.4 (0.1–835.8)	69.3 (0.4–849.6)	-1.8 (-49.6–21.6)	19.3 (0.002–422.1)	2.3 (0.0–128.9)
Research institute	17	227.2 (8.7–1146.0)	223.4 (11.4–1285.8)	229.1 (14.1–1318.4)	-5.7 (-32.6–2.2)	88.5 (3.4–355.1)	10.1 (0.0–55.6)
Corporatized public	129	56.5 (0.05–611.6)	54.9 (0.04–541.3)	57.7 (0.05–551.7)	-2.8 (-28.2–4.4)	15.2 (0.04–154.8)	1.4 (0.0–24.1)
**Total**	**805**	**59.1 (0.05–1146.0)**	**68.7 (0.04–1285.8)**	**70.8 (0.05–1318.4)**	**-2.1 (-49.6–21.6)**	**20.1 (0.002–422.1)**	**2.4 (0.0–128.9)**

Table 3. Spearman correlation coefficients (n=805).

Variable / Correlation indicator	Total assets (PLN)	Total revenues (PLN)	Total costs (PLN)	Gross profit/loss (PLN)	Total liabilities (PLN)	Arrears (PLN)	Gross profit margin (%)	Debt ratio (%)	Share of arrears in total liabilities (%)	Share of staff costs in total costs (%)
Total assets (PLN)	1,000	.924**	.921**	-.249**	.843**	.347**	-.231**	.164**	.276**	-.615**
Total revenues (PLN)	.924**	1,000	.998**	-.319**	.901**	.418**	-.300**	.371**	.347**	-.528**
Total costs (PLN)	.921**	.998**	1,000	-.356**	.911**	.435**	-.341**	.391**	.365**	-.528**
Gross profit/loss (PLN)	-.249**	-.319**	-.356**	1,000	-.453**	-.526**	.895**	-.479**	-.497**	.131**
Total liabilities (PLN)	.843**	.901**	.911**	-.453**	1,000	.599**	-.452**	.637**	.529**	-.515**
Arrears (PLN)	.347**	.418**	.435**	-.526**	.599**	1,000	-.510**	.614**	.982**	-.234**
Gross profit margin (%)	-.231**	-.300**	-.341**	.895**	-.452**	-.510**	1,000	-.519**	-.508**	.119**
Debt ratio (%)	.164**	.371**	.391**	-.479**	.637**	.614**	-.519**	1,000	.588**	-.072*
Share of arrears in total liabilities (%)	.276**	.347**	.365**	-.497**	.529**	.982**	-.508**	.588**	1,000	-.189**
Share of staff costs in total costs (%)	-.615**	-.528**	-.528**	.131**	-.515**	-.234**	.119**	-.072*	-.189**	1,000

*p<0.05; **p<0.0.001.

Table 4. Univariate logistic regression models predicting gross profit and arrears generation (including ownership groups).

Variable.	Generation/existence of gross profit		Generation/existence of arrears	
	**Adjusted OR (95% CI)**	**p - value**	**Adjusted OR (95% CI)**	**p - value**
**Ownership group:**				
country or city	1		1	
voivodeship	1.40 (0.99–1.96)	0.055	0.49 (0.35–0.70)	<0.001
medical university	1.01 (0.53–1.97)	0.968	0.98 (0.71–2.36)	0.610
ministry	2.79 (1.70–4.59)	<0.001	0.40 (0.21–0.67)	<0.001
**Assets (10 million PLN)**	0.94 (0.91–1.97)	<0.001	1.05 (1.03–1.07)	<0.001
**Revenues (10 million PLN)**	0.94 (0.92–0.96)	<0.001	1.06 (1.04–1.08)	<0.001
**Costs (10 million PLN)**	0.94 (0.91–0.96)	<0.001	1.06 (1.04–1.08)	<0.001

OR – odds ratio.

Table S5. Univariate logistic regression models predicting gross profit and arrears generation (including organisational form groups).

**Variable**	Generation/existence of gross profit		Generation/existence of arrears	
	**Adjusted OR (95% CI)**	**p - value**	**Adjusted OR (95% CI)**	**p - value**
**Organisational form:**				
SPZOZ	1		1	
Research institute	0.20 (0.06–0.70)	0.012	3.71 (1.29–10.64)	0.015
Corporatized public	0.50 (0.34–0.74)	0.001	1.08 (0.73–1.58)	0.710
**Assets (10 million PLN)**	0.95 (0.93–0.97)	<0.001	1.04 (1.02–1.06)	<0.001
**Revenues (10 million PLN)**	0.95 (0.93–0.96)	<0.001	1.06 (1.04–1.08)	<0.001
**Costs (10 million PLN)**	0.94 (0.92–0.96)	<0.001	1.06 (1.04–1.08)	<0.001

OR – odds ratio

Table S6. Multivariable logistic regression models predicting gross profit and arrears generation (ownership group and total assets as variables).

**Variable**	**Generation/existence of gross profit**		**Generation/existence of arrears**	
	**Adjusted OR (95% CI)**	**p - value**	**Adjusted OR (95% CI)**	**p - value**
**Ownership group:**				
country or city	1		1	
voivodeship	1.70 (1.19–2.42)	<0.001	0.41 (0.28–0.59)	<0.001
medical university	2.21 (1.05–4.65)	<0.001	0.61 (0.29–1.26)	
ministry	3.75 (2.19–6.43)	<0.001	0.31 (0.18–0.53)	<0.001
**Assets (10 million PLN)**	0.93 (0.91–0.96)	<0.001	1.06 (1.04–1.09)	<0.001

**AUC**	0.68 (0.64–0.72)	0.69 (0.65–0.73)

OR – odds ratio, AUC – area under the curve.

Table S7. Multivariable logistic regression models predicting gross profit and arrears generation (organizational form and total assets as variables).

Variable	Generation/existence of gross profit		Generation/existence of arrears	
	Adjusted OR (95% CI)	p - value	Adjusted OR (95% CI)	p - value
**Organisational form:**				
SPZOZ	1		1	
Research institute	0.33 (0.09–1.20)	0.092	2.34 (0.78–7.08)	0.131
Corporatized public	0.49 (0.33–0.72)	<0.001	1.08 (0.73–1.59)	0.705
**Assets (10 million PLN)**	0.95 (0.93–0.97)	<0.001	1.04 (1.02–1.06)	<0.001
**AUC**	0.67 (0.62–0.69)		0.67 (0.63–0.70)	

OR – odds ratio, AUC – area under the curve

Table S8. Multivariable logistic regression models predicting gross profit and arrears generation (ownership group and total costs as variables).

Variable	Generation/existence of gross profit		Generation/existence of arrears	
	Adjusted OR (95% CI)	p - value	Adjusted OR (95% CI)	p - value
**Ownership group:**				
country or city	1		1	
voivodeship	1.70 (1.19–2.42)	<0.001	0.37 (0.25–0.54)	<0.001
medical university	4.64 (1.95–11.01)	<0.001	0.28 (0.12–0.66)	<0.001
ministry	3.31 (1.94–5.65)	<0.001	0.34 (0.19–0.58)	<0.001
**Costs (10 million PLN)**	0.92 (0.90–0.84)	<0.001	1.08 (1.06–1.11)	<0.001
**AUC**	0.72 (0.68–0.75)		0.72 (0.68–0.76)	

OR – odds ratio, AUC – area under the curve.

Table S9. Multivariable logistic regression models predicting gross profit and arrears generation (organizational form and total costs as variables).

Variable	Generation/existence of gross profit		Generation/existence of arrears	
	Adjusted OR (95% CI)	p - value	Adjusted OR (95% CI)	p - value
**Organisational form:**				
SPZOZ	1		1	
Research institute	0.32 (0.09–1.19)	0.088	2.26 (0.73–6.98)	0.156
Corporatized public	0.45 (0.30–0.68)	<0.001	1.16 (0.79–1.73)	0.453
**Costs (10 million PLN)**	0.94 (0.92–0.96)	<0.001	1.06 (1.04–1.08)	<0.001
**AUC**	0.70 (0.66–0.74)		0.72 (0.68–0.75)	

OR – odds ratio, AUC – area under the curve.
